# A phase II study of capecitabine and irinotecan in combination with concurrent pelvic radiotherapy (CapIri-RT) as neoadjuvant treatment of locally advanced rectal cancer

**DOI:** 10.1038/sj.bjc.6603645

**Published:** 2007-02-27

**Authors:** F Willeke, K Horisberger, U Kraus-Tiefenbacher, F Wenz, A Leitner, A Hochhaus, R Grobholz, A Willer, G Kähler, S Post, R-D Hofheinz

**Affiliations:** 1 Chirurgische Klinik, Medizinische Fakultät Mannheim der Universität Heidelberg, Mannheim, Germany; 2 Klinik für Strahlentherapie und Radioonkologie, Medizinische Fakultät Mannheim der Universität Heidelberg, Mannheim, Germany; 3 Onkologisches Zentrum, III. Medizinische Klinik, Medizinische Fakultät Mannheim der Universität Heidelberg, Mannheim, Germany; 4 Institut für Pathologie, Medizinische Fakultät Mannheim der Universität Heidelberg, Mannheim, Germany

**Keywords:** capecitabine, irinotecan, locally advanced rectal cancer, chemoradiotherapy

## Abstract

We sought to evaluate the efficacy and safety data of a combination regimen using weekly irinotecan in combination with capecitabine and concurrent radiotherapy (CapIri-RT) as neoadjuvant treatment in rectal cancer in a phase-II trial. Patients with rectal cancer clinical stages T3/4 Nx or N+ were recruited to receive irinotecan (50 mg m^−2^ weekly) and capecitabine (500 mg m^−2^ bid days 1–38) with a concurrent RT dose of 50.4 Gy. Surgery was scheduled 4–6 weeks after the completion of chemoradiation. A total of 36 patients (median age 62 years; m/f: 27:9) including three patients with local recurrence were enclosed onto the trial. The median distance of the tumour from the anal verge was 5 cm. The main toxicity observed was (NCI-CTC grades 1/2/3/4 (*n*)): Anaemia 23/9/−/−; leucocytopenia 12/7/7/2, diarrhoea 13/15/4/−, nausea/vomiting 9/10/2/−, and increased activity of transaminases 3/3/1/−. One patient had a reversible episode of ventricular fibrillation during chemoradiation, most probably caused by capecitabine. The relative dose intensity was (median/mean (%)): irinotecan 95/91, capecitabine 100/92). Thirty-four patients underwent surgery (anterior resection *n*=25; abdomino-perineal resection *n*=6; Hartmann's procedure *n*=3). R0-resection was accomplished in all patients. Two patients died in the postoperative course from septic complications. Pathological complete remission was observed in five out of 34 resected patients (15%), and nine patients showed microfoci of residual tumour (26%). After a median follow-up of 28 months one patient had developed a local recurrence, and five patients distant metastases. Three-year overall survival for all patients with surgery (excluding three patients treated for local relapse or with primary metastatic disease) was 80%. In summary, preoperative chemoradiation with CapIri-RT exhibits promising efficacy whereas showing managable toxicity. The local recurrence and distant failure rates observed after a median 28 months are low compared with standard 5-fluorouracil based therapy.

Advances in surgical technique, including total mesorectal excision and thorough pathohistological work-up of the resected specimen have significantly improved the prognosis of patients with localised rectal cancer during the past two decades. Nevertheless, preoperative radiotherapy further improves local recurrence rates (Kapiteijn *et al*, 2001). The German CAO/AIO/ARO-94 trial, which compared pre- and postoperative chemoradiation in resectable rectal cancer, established the neoadjuvant approach as a new standard of care given it's superiority with respect to local failure rates and toxicity (Sauer *et al*, 2004). Moreover, the MRC CR07 trial, which compared short-course preoperative radiotherapy (5 × 5 Gy) in resectable rectal cancer with selective postoperative chemoradiation in patients with compromised circumferential resection margins, showed a significant reduction in local recurrence and improved disease-free survival in favour of the short-course preoperative radiotherapy (Sebag-Montefiore *et al*, 2006). Finally, both the EORTC 22921 and the FFCD 9203 trials demonstrated that adding 5-fluorouracil (5-FU) to preoperative radiotherapy further diminishes the rate of local recurrence and increases the rate of pathological complete remissions (pCR) (Bosset *et al*, 2005; Gerard *et al*, 2005). Thus, preoperative 5-FU-based chemoradiation has meanwhile gained a high level of evidence in the treatment of resectable rectal cancer. Moreover, data from several single-agent chemoradiation studies provide a clear rationale for a simplification of chemoradiotherapy by the replacement of infusional 5-FU with the oral prodrug capecitabine (Xeloda, Hoffmann-La Roche Inc., Basel, Switzerland) (reviewed by Glynne-Jones *et al*, 2006).

Local recurrence appears to be now less of a problem, whereas distant metastasis remains the most common form of treatment failure. Intensified neoadjuvant chemoradiation using two drug-schedules are under investigation mainly for two reasons: (i) it is hoped that early administration of systemically effective doses of chemotherapy might improve long-term outcome and reduce the rate of distant failure by the early treatment of micrometastases; (ii) intensified two drug regimen increase, the likelihood of tumour remission (especially the pCR rate) (Hartley *et al*, 2005) which might augment the chance to obtain clear circumferential margins. Moreover, there is a strong evidence that pCR is a prognostic factor for patients undergoing neoadjuvant chemoradiation for rectal cancer (Roh *et al*, 2004; Rödel *et al*, 2005).

The feasibility and efficacy of combinations of 5-FU or capecitabine with newer drugs like irinotecan (Campto, Pfizer, Karlsruhe, Germany) during neoadjuvant chemoradiotherapy has been demonstrated in several trials (reviewed by Klautke *et al*, 2005). Irinotecan has radiosensitizing properties (reviewed by Mitchell, 2000) and is a standard for care in the first-line treatment of metastatic colorectal cancer (eg Köhne *et al*, 2005). In the phase-I trial, we established a chemoradiotherapy regimen adding weekly irinotecan 50 mg m^−2^ and capecitabine (500 mg m^−2^ bid) to conventionally fractionated radiotherapy (CapIri-RT) for the neoadjuvant treatment of rectal cancer (Hofheinz *et al*, 2005). The tolerability was good and preliminary pCR rates were encouraging.

The present phase-II trial sought to expand the safety and efficacy data obtained in the dose-finding trial. Here we report on the acute toxicity and efficacy of the CapIri-RT regimen along with the follow-up data of 36 patients.

## PATIENTS AND METHODS

### Eligibility criteria

Patients with histologically confirmed locally advanced non-metastastic rectal adenocarcinoma (cT3/4 or cTxN+) were eligible for entry in the study. The definition of the T category was made by transrectal ultrasonography and pelvic computed tomographic (CT) scans. Further inclusion criteria comprised: Eastern Cooperative Oncology Group (ECOG) performance score ⩽2; age ⩾18 years; adaequate bone marrow function (leucocyte count >3000 *μ*l^−1^, platelet count >100 000 *μ*l^−1^); and adaequate renal (serum creatinine ⩽1.4 mg dl^−1^ or creatinine clearance >60 ml min^−1^) and hepatic function (bilirubin ⩽2 mg dl^−1^). Patients with a history of Gilbert-Meulengracht's disease were excluded. Patients of childbearing potential were required to use appropriate contraception. Patients were excluded if they suffered from other cancers, ischaemic heart disease or had known hypersensitivity to 5-FU and/or irinotecan.

The protocol was reviewed and approved by the local institutional review board and the study was performed according to the Declaration of Helsinki. All patients provided written informed consent before entering the trial.

### Pretreatment evaluation

Before study admission, all patients underwent a complete history, physical examination, biopsy with confirmation of adenocarcinoma, digital rectal examination, rectoscopy, transrectal ultrasonography, pelvic and abdominal CT scans, colonoscopy, and chest X-rays. Since the study protocol derived from 2002, when MRI imaging has not yet found widespread use in Germany, MRI staging was not mandatory in this study. A full blood count with differential and serum chemistry (including electrolytes, creatinine, urea, uric acid, transaminases, total bilirubin, alkaline phosphatase, and lactic dehydrogenase) was obtained within 14 days before the start of treatment. Weekly blood counts were obtained, and serum chemistry was repeated every third week or whenever clinically indicated.

### Radiotherapy

Radiotherapy (RT) was delivered with a linear accelerator using 23 MeV photons and a three-field box technique consisting of a posterior–anterior and two lateral fields. For three-dimensional treatment planning purposes, all patients had a CT scan in the treatment position (prone position) using a belly board. A total irradiation dose of 50.4 Gy was given in daily fractions of 1.8 Gy, 5 days a week. The clinical target volume (CTV) according to the ICRU included the sacrum, the praesacral space and the posterior wall of the bladder, and prostate/vagina. The common iliac lymph nodes were included in the CTV. The upper border of the CTV was at the L5/S1 interspace for cN0 and at L4/L5 for cN+ patients. The lower field border was 5 cm below the macroscopic tumour. After a dose of 45 Gy the small bowel was excluded and an additional dose of 5.4 Gy was given to boost the volume using a shrinking field technique (days 36–38).

### Chemotherapy

Starting at day 1 of RT, patients received capecitabine 500 mg m^−2^ orally bid within 30 min after a meal (generally after breakfast and dinner) for the whole time of irradiation (days 1–38), and weekly irinotecan 50 mg m^−2^ beginning on day 1 of RT for 5 consecutive weeks (days 1, 8, 15, 22, and 29) (Hofheinz *et al*, 2005). All patients received standard antiemetic prophylaxis using 5-HT_3_ antagonists and dexamethasone.

If a patient reported one of the following adverse events according to National Cancer Institute common toxicity criteria (NCI-CTC version 3.0), chemotherapy was interrupted until adverse events had resolved to grade 0 or 1: leucocytopenia ⩾grade 2, thrombocytopenia ⩾grade 2, diarrhoea ⩾grade 2, mucositis/stomatitis ⩾grade 2, skin adverse events ⩾grade 2, and other adverse events >grade 2. Treatment was resumed in these cases with a dose reduction of 25% for both drugs (in case of hand-foot skin reaction only capecitabine was reduced by 25%). On the second occurrence of an adverse event ⩾grade 2, the capecitabine and irinotecan dose was to be reduced to 50% of the starting dose (except in the case of, hand-foot skin reaction; only capecitabine was to be reduced by 50%).

The application of postoperative adjuvant chemotherapy was left at the discretion of the treating physician.

All effort was made to continue daily RT without interruption, unless the patient experienced persistent or severe adverse event. The RT schedule was not modified or interrupted, unless an adverse event (such as a skin adverse event ⩾grade 2) was related to radiation. In these cases, RT was withheld until adverse events resolved to grade 0 or 1.

### Surgery and histopathologic examination of resected specimen

Surgical resection (either low anterior or abdomino-perineal resection) with total mesorectal excision (TME) was scheduled 4–6 weeks after completion of CapIri-RT. A time interval of 4–6 weeks between the termination of chemoradiotherapy and surgery was chosen, because it is considered a standard of care by most German centers. Pathological examination of the surgical specimen involved opening the specimen, identification of the former tumour-bearing area and macroscopic description followed by fixation in 10% neutral-buffered formalin for 24 h. After fixation, specimens were processed according to standard protocols (Compton, 2000). For obvious residual tumour, at least four paraffin blocks were processed. If no obvious residual tumour was present, the whole former tumour-bearing area was sliced and embedded. The completion of TME, circumferential resection margins, proximal and distal tumour-free margins, tumour mass, fibrotic changes, and irradiation vasculopathy were evaluated and semiquantitatively described whenever feasible.

### Study design and data evaluation

The primary end point of this phase II study was to assess the toxicity, efficacy (especially the pCR rate), and surgical morbidity of the CapIri-RT regimen (capecitabine 500 mg m^−2^ bid and weekly irinotecan 50 mg m^−2^ in combination with standard radiotherapy with 50.4 Gy (Hofheinz *et al*, 2005)). Secondary end points comprise the assessment of the rate of local and distant failure.

The statistical calculation was made on toxicity issues and was based on the definition of dose-limiting toxicity (DLT) used in our previous phase-I trial (Hofheinz *et al*, 2005). Dose-limiting toxicity had been defined in this phase-I trial by the occurrence of one of the following adverse events: grade 4 leukocytopenia/neutropenia or thrombocytopenia, symptomatic thrombocytopenia, grade 3/4 febrile neutropenia, or any ⩾grade 3 non-haematological adverse events except renal adverse events (⩾grade 2) and nausea/vomiting.

The rate of DLTs during the present phase-II trial should be lower than 17% (ie about one out of six patients). With a total of 32 patients, a rate of at least 83% of the patients experiencing no DLTs during radiochemotherapy can be assumed using Simon's two-stage design with a power of 80% and an *α*-value of 0.2 if less than five out of 15 (first stage) or eight out of 32 patients (second stage) experienced DLTs.

Deadline for the follow-up data evaluation was 31 May 2006. Survival according to Kaplan–Meier was performed using the Graph-Pad Prism software 4.0 (GraphPad Software, San Diego, CA, USA). Survival was calculated from the start of study treatment until patients death or the date of last follow-up. Progression free-survival was calculated from the start of treatment until the detection of local recurrence or distant metastases.

## RESULTS

Out of 36 patients entering this single-center trial, 33 had newly diagnosed rectal adenocarcinoma, and three patients had a local recurrence at the anastomotic site (Table 1). None of these patients had received prior pelvic radiotherapy. The median distance from the lower border of the primary to the anal verge was 5 cm (2–15 cm). Most tumours were located in the lower third of the rectum (*n*=21 patients). Only five patients had tumours in the upper third.

Two patients with potentially resectable hepatic metastases and one patient with suspected pleural carcinomatosis (not histologically verified) were included in this trial.

### Toxicity and dose intensity

Acute toxicity during chemoradiotherapy is listed in Table 2. The most frequently observed haematologic adverse event was leucocytopenia. Nine out of 36 patients had grade 3 or 4 leukocytopenia (25%), but no episode of neutropenic fever was reported. No patient received prophylactic or therapeutic granulocyte colony-stimulating factors during the study. Mild thrombocytopenia was only seen in two patients. The median decrease of haemoglobin level was 2.1 g dl^−1^ (ranging from 0.0 to 3.5 g dl^−1^), but grade 3 or 4 anaemia or major bleeding was not observed. Of note, no prophylactic measures (eg recombinant human erythropoetin) were in place and no blood transfusions were required during chemoradiotherapy.

Gastrointestinal adverse events were observed more frequently. Fifteen patients reported diarrhoea grade 2 and four had diarrhoea grade 3. The median duration of diarrhoea grade 3 was 6 days (range 3–8 days). Nausea and vomiting grade 3 was observed in two patients. With respect to laboratory values, increased activity of transaminases was noted in seven patients and one patient developed hyperbilirubinaemia grade 2. Moreover, this latter patient developed grade 4 leucocytopenia, long-lasting diarrhoea grade 3 over 8 days with marked abdominal cramping and alopecia. Screening for mutations of the dihydropyrimidine dehydrogenase enzyme was inconspicuous, but molecular testing revealed homozygosity for an aberrant UGT1A1 promoter (TA)_7/7_, genotype. This disorder is associated with reduced UGT enzyme activity, impared Sn-38 metabolism, and substantially increased irinotecan toxicity. Interestingly, this patient had a normal ‘phenotype’ without a history of icterus intermittens (Gilbert-Meulengracht) or elevation of bilirubine baseline values.

Hand-foot skin reaction was rarely observed owing to the low doses of capecitabin used and slight alopecia comprised five patients. Radiation-induced dermatitis affected a total of 19 patients, seven of who had grade 2.

A 42-year-old male patient had a reversible episode of ventricular fibrillation during physical exercises in the fifth week of chemoradiation. The resuscitation measures were immediately successful. Coronary heart disease and electrophysical disorders were excluded by heart catheterisation. The patient received an internal implantable heart rhythm converter and defibrillator. Chemotherapy was stopped, but radiotherapy was continued until 50.4 Gy. We speculate that this episode was related to a coronary spasm due to capecitabine treatment. Interestingly, no further episodes of ventricular fibrillation were recorded till now.

Four patients had to be hospitalised owing to diarrhoea for a median of 7 days (range 6–16 days). Radiotherapy was delivered till a dose of 50.4 Gy in all but five patients. Radiotherapy was terminated in these patients at 39.6 Gy (*n*=1), 45 Gy (*n*=3), and 48.6 Gy (*n*=1) owing to severe fatigue grade 3 (*n*=1), diarrhoea grade 3 (*n*=2), and nausea grade 2 and 3 (*n*=2).

In all, a total of seven patients developed DLTs according to prespecified criteria (see Study design and data evaluation). Thus, a true rate of 83% of the patients experiencing no DLTs during radiochemotherapy can be assumed with a power of 80% and an *α*-value of 0.2.

A median 100% of the scheduled capecitabine dose (range 45–100%; mean 92%) and a median 95% of the scheduled irinotecan dose (range 60–100%; mean 91%) was applied. Chemoradiotherapy was delivered as scheduled in 18 patients, whereas 12 patients came in need of postponement of chemotherapy (7 days delay *n*=8 patients; 8–14 days *n*=4 patients) but received radiotherapy as scheduled. As mentioned earlier, one patient was withdrawn from chemotherapy owing to cardiotoxicity but radiotherapy continued, and chemoradiotherapy was terminated in five patients for toxicity reasons.

### Surgery and pathological features of the resected specimen

Radical TME surgery was scheduled 4–6 weeks after the termination of the CapIri-RT treatment. Two patients did not proceed to surgery owing to the presence of metastatic disease detected immediately before resection (*n*=1) and patient refusal (*n*=1). Median time to surgery calculated from the last day of chemoradiation was 4.7 weeks (range 2.4–11.0 weeks). All but one patient, who requested surgery after 2.4 weeks owing to personal reasons, underwent resection at least 4 weeks after the termination of chemoradiotherapy. Of the 34 surgical procedures, 25 patients were anterior resections (74%), six patients required abdomino-perineal resection (18%), and three underwent a Hartmann's procedure (8%). Of 23 patients with tumours located in the lower third of the rectum, 13 had sphincter sparing surgery (57%). Table 3 illustrates the type of resection in relation to the location of the primary tumour. All 34 patients had R0-resection with clear circumferential margins.

Postoperative morbidity comprised prolonged/complicated wound healing (*n*=9; 26%), temporary bowel atonia (*n*=8; 24%), anastomotic leakage (*n*=3; 12%), and abscess (*n*=3; 9%). Two patients died postoperative owing to septic complications following anastomotic leakage and pneumonia. Surgery took place on days 50 and 56 after the termination of chemoradiotherapy. Of these, one patient developed a systemic inflammatory syndrome (MRSA infection with endocarditis and pneumonia) and another patient had acute coronary syndrome, aspiration and ARDS.

A complete regression of the tumour (pCR; ypT0 N0) was found in five (15%), and microfoci (few tumour cells scattered within fibrotic tissue) were found in another nine out of 34 resected patients. A median of 14 and a total of 456 lymph nodes were examined. Eleven patients had positive lymph nodes (N1-status *n*=10; N2-status *n*=1). Nineteen out of 30 patients with positive lymph nodes in pretreatment diagnostics were lymph node negative on pathological examination. T category was downstaged in 18 of 33 evaluable patients (55%) (Table 4). The pre-treatment assessment of the T category was made by endoscopic ultrasound and CT scans, since MRI staging had not yet become a standard of care in Germany when the present study concept was designed.

#### Follow-up, local recurrence and survival

The median follow-up is 26.3 months for all 36 patients and 27.6 months (range 16–48 months) for surviving patients.

Thirty-six patients entered the study, of whom 34 proceeded to surgery. Two of these died postoperatively and three patients underwent resection of the primary but had irresectable liver or pleural metastases which were either deemed resectable upon study entry or uncertain in CT scan. Thus, a total of 29 patients were followed-up after potentially curative resection. Of these, three patients (10.3%) developed distant metastases (lung and/or pleura *n*=2; lymph nodes *n*=1). One single patient developed local recurrence after 15 months and underwent salvage surgery. Of note, this patient had a ypT4N2 tumour after primary surgery.

Considering all patients included in this trial, nine out of 36 patients (25%) have died, of whom four succumbed tumour-related. Among these, two patients had metastases at the time of diagnosis and one patient had refused potentially curative sugery. Three patients died non-tumour related (17.7, 29.5, and 39.5 months after the start of therapy).

Considering all patients (*n*=36) actuarial calculated overall survival is 83% at 2 years (patients at risk *n*=23), and 78% at 2.5 years (patients at risk *n*=15).

## DISCUSSION

Several trials on perioperative therapy in rectal cancer have significantly contributed to a better understanding of an optimized therapeutic strategy during the past few years. It could be demonstrated that (i) the best way to deliver radiotherapy is neoadjuvant (CAO/AIO-ARO-94 trial (Sauer *et al*, 2004; MRC CR07 – Sebag-Montefiore *et al*, 2006), (ii) adding 5-FU to neoadjuvant radiotherapy improves the pCR and the local recurrence rates albeit by the price of higher acute toxicity (EORTC 22921 – Bosset *et al*, 2005; FFCD 9203 – Gerard *et al*, 2005), (iii) postoperative 5-FU based adjuvant therapy might further improve disease-free survival (n.s.) (EORTC 22921 – Bosset *et al*, 2005). Nevertheless, none of these strategies has either decreased the rate of distant metastases or led to improved survival results.

One strategy to improve overall survival and decrease the rate of distant failure is the intensification of preoperative chemoradiation. By using several drugs during neoadjuvant radiotherapy it is hoped that the local R0-resection rate further increases and that a higher amount of distant metastases is eradicated at the earliest time point. Intensification of postoperative chemotherapy in early-stage *colon* cancer patients has already proven to ameliorate the results of adjuvant treatment. Both the MOSAIC and the NSABP C-07 trial have unequivocally demonstrated an improved 3-year disease free survival (DFS) using adjuvant oxaliplatin in combination with 5-FU/FA regimen for the adjuvant treatment instead of using 5-FU/FA alone (André *et al*, 2004; Wolmark *et al*, 2005). Moreover, the PETACC 3 study reported a 3-year DFS advantage for an irinotecan/5-FU/FA regimen as well, although this study did not reach statistical significance, most probably because of an to an imbalance in risk factors in both treatment arms (van Cutsem *et al*, 2005).

In the present phase-II trial we investigated a previously established regimen using daily capecitabine and weekly irinotecan in conjunction with 50.4 Gy (CapIri-RT – Hofheinz *et al*, 2005). A total of 36 patients were treated with this regimen, of whom 34 proceeded to surgery. We found a considerable rate of pCR and microfoci (14 out of 34 resected patients; 41%), which appears to be almost doubled in comparison to 5-FU-based chemoradiotherapy regimen provided that standardised histopathological work-up is in place. This finding is consistent with data published by Klautke *et al* (2006) using a CapIri-RT regimen with somewhat higher doses of capecitabin (750 mg m^−2^ bid) and irinotecan (6 × weekly 40 mg m^−2^). A total of seven out of 26 evaluable patients in this phase I/II trial had pCR or microfoci. Moreover, using 5-FU and irinotecan, Klautke *et al* (2005) had previously observed a rate of 50% of pCR or microfoci in 36 patients undergoing neoadjuvant chemoradiation. Another trial from Wales and England – thus far only published as abstract – confirmes the feasibility of the CapIri-RT regimen using capecitabine 650 mg m^−2^ in conjunction with irinotecan 60 mg m^−2^ 4 times weekly (Gollins *et al*, 2006).

With respect to acute toxicity the primary concern of capecitabine/irinotecan combinations is diarrhoea. The rate of grade 3–4 diarrhoea reported in the CapIri-RT trials is about 15–30% (Hofheinz *et al*, 2005; Gollins *et al*, 2006; Klautke *et al*, 2006). Using capecitabine and oxaliplatin combinations, grade 3–4 diarrhoea is reported to range between 18% (*n*=85; CORE-trial; Rutten *et al*, 2006) and 30% (*n*=40; RadiOxCape-study; Machiels *et al*, 2005). In this cross-trial comparison neither CapIri-RT or CapOx-RT regimen seem to be advantageous although the use of different chemotherapy regimen, radiotherapy doses and a limited number of patients included in these trials preclude firmer conclusions. The only randomised trial, – published as an interim analysis at the ASCO meeting 2006, included a total of 35 patients and used (i) induction chemotherapy with CapOx and CapIri and (ii) very high doses of irinotecan during radiochemotherapy (180 mg m^−2^), which might not be suited to exploit the radiosensitizing properties of irinotecan (Privitera *et al*, 2006).

Major interest should be paid to the rate of surgical complications after intensified chemoradiotherapy using irinotecan- or oxaliplatin-based intensified chemoradiotherapy. The limited data available on surgical morbidity after neoadjuvant intensified chemoradiation give the impression of an increased rate of anastomotic leakage by the use of combination regimens. Of course, trials randomizing between 5-FU- or capecitabine-based chemoradiation and intensified regimen using additional irinotecan or oxaliplatin are mandatory to assess the extent of surgical or late morbidity caused by intensified chemoradiotherapy regimen. In the present study, we observed a rate of 12% of anastomotic leakage after deep-anterior resection. Although this rate almost exactly matches the anastomotic leakage rate in the neoadjuvant chemoradiotherapy arm of the German rectal cancer trial using 5-FU alone (Sauer *et al*, 2004) it appears to be high in a single-centre trial. Clearly, this could be owing to a limited number of patients in the study. Nonetheless, a thorough reporting of surgical complications in all studies using intensified chemoradiotherapy regimen appears to be of great interest. One might speculate that the price paid for a higher rate of pCR or microfoci obtained by intensified regimens is a higher risk of surgical morbidity, for example, anastomotic leakage. We therefore, advocate for a detailed reporting of surgical morbidity in all trials on intensified chemoradiotherapy.

With regard to long-term outcome, we observed only one local recurrence (a patient with ypT4N2 at surgery) after a median observation time of 28 months in surviving patients, suggesting a high rate of local control. Five patients developed distant metastases. The actuarial 2-year survival for all 36 patients is 83% (2.5-year survival 78%). Similarly, data on 36 patients receiving irinotecan/5-FU-based chemoradiotherapy published after a median follow-up of 40 months suggest that irinotecan-based regimen might provide superior long term results (4-year local recurrence rate 7% and disease-specific survival of 70%; Klautke *et al*, 2005). Clearly, larger (randomized) trials and longer follow-up periods are needed.

In all, the CapIri-RT regimen appears to be safe resulting in a rate of about 40% of patients with pCR or microfoci at surgery. Larger trials and longer follow-up are awaited in order to assess the real impact of the higher remission rate on long-term results. Another important issue to be adressed in prospective trials is, whether or to which extent intensified regimen compromise the safety of surgery. After years of stagnation in the treatment of rectal cancer intensified neoadjuvant and/or adjuvant combination chemo(radio)therapy regimens using newer cytostatics and maybe targeted therapies clearly hold the promise to further improve the treatment of this disease.

## Figures and Tables

**Table 1:** Patient and tumour characteristics

**No. of patients**	**36**
Median age (range)	62 years (36–82)
	
*Gender*	
Male	27
Female	9
	
*ECOG performance status*	
0	15
1	18
2	3
	
*Clinical staging*	
T2	4
T3	26
T4	5
n.a.	1
cN 0	4
cN +	31
n.a.	1
	
*Distant metastases*	
Resectable (liver)	2
Supected (pleura, irresectable)	1
	
*Distance from lower tumour margin to anal verge*	
Median	5 cm
Range	2–15 cm
0–5 cm	21
>5–10 cm	9
>10 cm	5
NA	1

**Table 2:** Incidence and maximum toxicity observed during CapIri-RT treatment (*n*=36 patients)

	**Grade 1**	**Grade 2**	**Grade 3**	**Grade 4**
*Haematologic*				
Leucocytopenia	12	7	7	2
Thrombocytopenia	2	—	—	—
Anaemia	23	9	—	—
				
*Gastrointestinal*				
Nausea/vomiting	9	10	2	—
Mucositis/stomatitis	2	—	—	—
Diarrhoea	13	15	4	—
Proctitis	11	4	—	—
Abdomial cramping	6	4	—	—
				
*Laboratory*				
Hyperbilirubinaemie	2	1	—	—
ALAT/ASAT elevation	3	3	1	—
Creatinine elevation	3	—	—	—
				
*Skin*				
Hand-foot skin reaction	3	—	—	—
Alopecia	5	—	—	—
Radiation dermatits	12	7	—	—
				
*Other*				
Fatigue	11	6	1	—
Cholinergic syndrome	2	—	—	—

CTC, common toxicity criteria; NCI, National Cancer Institute.

Adverse events are graded according to CTC of the NCI version 2.0.

**Table 3:** Surgical approach and tumour distance from the anal verge (*n*=34)

	**Surgical approach (no. of patients)**			
**Tumour distance from anal verge**	**Deep anterior resection**	**Abdomino-perineal resection**	**Hartmann resection**	**Total**
0–5 cm	13	6	2	21
>5–10 cm	7	0	1	8
>10 cm	5	0	0	5
All tumours	25	6	3	34

Note: The tumor distance could not be calculated in one patient with local relapsed rectal cancer, and one patient refused surgery.

**Table 4:** Pathological staging (ypT) compared with clinical staging (cT) at baseline (*n*=33)

	**No. of patients**					
	**ypT0**	**ypT1**	**ypT2**	**ypT3**	**ypT4**	**yN+**
cT2 (*n*=4)	—	2	1	1	—	
cT3 (*n*=26)	4	3	6	11	2	
cT4 (*n*=3)	1	—	2	—	—	
cN+ (*n*=30)						11
cN− (*n*=3)						0

Note: Clinical T- or N- staging was not evaluable in one patient each. Two patients (including one patient with indeterminable cN status) did not undergo surgery.
